# Survey of Antimicrobial-Resistant Bacteria Isolated from Rivers in Japan, Indonesia and Nepal

**DOI:** 10.3390/pathogens15030317

**Published:** 2026-03-15

**Authors:** Kayo Osawa, Ryohei Nomoto, Takashi Suzuki, Taishi Maeda, Ganesh Rai, Shouhiro Kinoshita, Noriko Nakanishi, Dadik Raharjo, Masanori Kameoka, Masato Fujisawa, Shiba Kumar Rai, Kuntaman Kuntaman, Toshiro Shirakawa

**Affiliations:** 1 Department of Medical Technology, Kobe Tokiwa University, Kobe 653-0838, Japan; t-suzuki@kobe-tokiwa.ac.jp (T.S.);; 2 Department of Infectious Diseases, Kobe Institute of Health, Kobe 650-0046, Japan; 3 Department of Medical Microbiology, Shi-Gan International College of Science and Technology, Kathmandu 44600, Nepal; 4 Division of Advanced Medical Science, Kobe University Graduate School of Science, Technology and Innovation, Kobe 650-0017, Japantoshiro@med.kobe-u.ac.jp (T.S.); 5 Institute of Tropical Disease, Airlangga University, Surabaya 60286, Indonesiakuntaman@fk.unair.ac.id (K.K.); 6 Department of Public Health, Kobe University Graduate School of Health Sciences, Kobe 654-0142, Japan; mkameoka@port.kobe-u.ac.jp; 7 Division of Urology, Department of Organ Therapeutics, Faculty of Medicine, Kobe University Graduate School of Medicine, Kobe 650-0017, Japan; 8 Department of Microbiology, Nepal Medical College Teaching Hospital, Kathmandu 44600, Nepal; 9 Department of Microbiology, Faculty of Medicine, Airlangga University, Surabaya 60132, Indonesia

**Keywords:** antimicrobial resistance (AMR), antimicrobial-resistant bacteria (ARB), antimicrobial resistance genes (ARGs), environmental river, One Health approach, selective culture–based Next Generation Sequencing analysis (scNGS analysis), resistome analysis

## Abstract

The threat of antimicrobial resistance in aquatic environments, particularly riverine systems, is escalating, in part due to effluents discharged from healthcare facilities. This issue has been recognized not only in Japan but also in other Asian countries such as Indonesia and Nepal. Nevertheless, existing research remains limited, prompting an investigation into the prevalence of antimicrobial-resistant bacteria in the upstream and downstream sites of environmental rivers. In 2024, six samples were collected from three rivers in Hyogo Prefecture, Japan; five samples from five river sites in Indonesia; and three samples from downstream sites of rivers in Kathmandu, Nepal. These samples were subjected to selective culture–based Next Generation Sequencing and resistome analyses, based exclusively on the selective culture of bacteria propagated on CHROMagar ESBL plates. In Japan and Indonesia, *Pseudomonas*, *Stenotrophomonas* and *Acinetobacter* were frequently detected, whereas *Klebsiella* was overwhelmingly predominant in Nepal. Significant differences in the similarity of bacterial community composition among sampling sites across the three countries were observed (*p* < 0.001). Notably, Nepal exhibited the highest abundance level of antimicrobial resistance genes among the three countries, largely consisting of β-lactam resistance genes. In conclusion, these analyses elucidated substantial differences in bacterial community composition and degrees of environmental contamination.

## 1. Introduction

Antimicrobial resistance (AMR), encompassing antimicrobial-resistant bacteria (ARB) and their associated resistance genes, is widely recognized as a critical global threat to human health [1,2]. Increasing concern has been directed toward the dissemination of ARB from environmental reservoirs, particularly aquatic ecosystems such as rivers. For instance, AMR may be introduced into natural water bodies through effluents released from healthcare facilities. Consequently, urban rivers are increasingly regarded as reservoirs and potential hotspots for the persistence and proliferation of ARB. In response to this challenge, the One Health approach has been actively promoted worldwide. The One Health paradigm emphasizes the necessity of coordinated actions across regional, national, and international sectors to achieve optimal health outcomes by preventing environmental contamination and mitigating health risks associated with ARB that circulate among humans, animals, and the environment [1,2,3,4]. Within this framework, particular attention has been devoted to extended-spectrum β-lactamase (ESBL)–producing bacteria and carbapenemase-producing bacteria [5,6]. A comprehensive elucidation of the distribution and dynamics of AMR among environmental microorganisms is essential for clarifying transmission pathways and informing effective mitigation strategies, and such investigations are expected to advance our understanding of the mechanisms underlying the spread of AMR. Previous investigations in Japan have predominantly concentrated on antimicrobial susceptibility testing of hospital-derived bacterial isolates and on the characterization of bacteria harboring antimicrobial resistance genes. However, we contend that studies confined exclusively to hospital settings are inherently limited in their capacity to elucidate the transmission dynamics of an ARB and antimicrobial resistance genes (ARGs) [7,8]. Furthermore, we have established research infrastructures not only in Japan but also in Indonesia and Nepal. To date, we have undertaken epidemiological investigations of urinary tract infections in Indonesian hospitals [9]. More recently, in addition to these clinical studies, we have examined ARB isolated from retail chicken meat [10]. Additionally, bacterial strains collected from six rivers in Indonesia were subjected to whole-genome sequencing and resistome analyses, revealing that ARBs are also widely disseminated in environmental settings [11]. In our previous work in Nepal, we conducted surveys of mosquito breeding habitats associated with vector-borne diseases, with particular emphasis on the aquatic environments supporting larval development. Building upon our prior analysis of these water sources, we were motivated to undertake a systematic assessment of their current environmental status [12]. In Japan, only a few reports have been published, primarily from areas near Yodogawa Rive (Osaka), Kanda River (Tokyo) and Yamanashi Prefecture [13,14,15]. In Nepal, an emerging country, recent studies have reported bacteria isolated from water sources [16,17]. However, such studies remain extremely limited, and comparative analyses with other countries are lacking. Across many Asian countries, including Japan, comprehensive surveys comparing ARGs in ARB derived from humans, animals (livestock), and environmental sources (rivers) remain notably insufficient. Furthermore, comparative assessments of samples collected from upstream and downstream sections of rivers have rarely been conducted. Therefore, in the present study, we investigated patterns of AMR by performing selective culture–based NGS analysis (scNGS analysis) and resistome analyses of bacterial communities—primarily β-lactam-resistant bacteria—isolated from water samples collected from both upstream and downstream river sites in Hyogo Prefecture, Japan, as well as from rivers in other Asian countries such as Indonesia and Nepal.

## 2. Materials and Methods

### 2.1. Sample Collection

**Figure 1:**
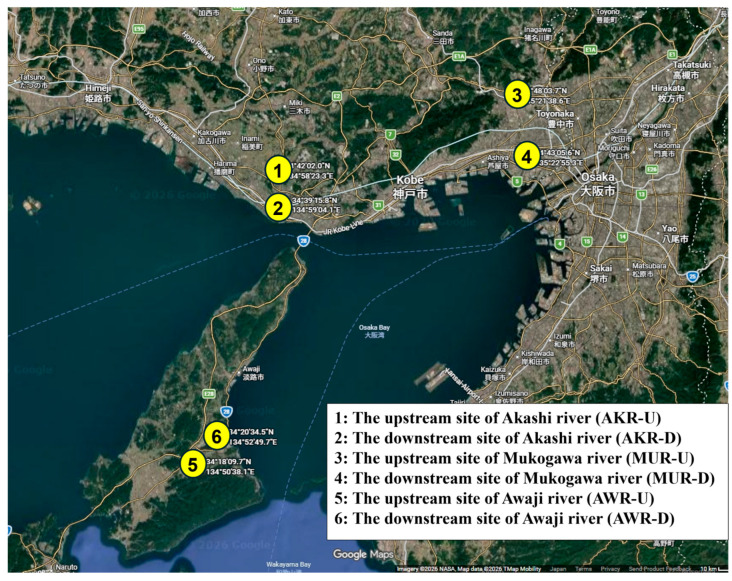
Map of six water sampling sites in three rivers in Hyogo Prefecture, Japan. Akashi River (upstream site: Akashi city, downstream site: Kobe city), Mukogawa River (upstream site: Takarazuka city, downstream site: Nishinomiya city), and Awaji River (upstream and downstream sites: Sumoto city in Awaji Island)].

**Figure 2:**
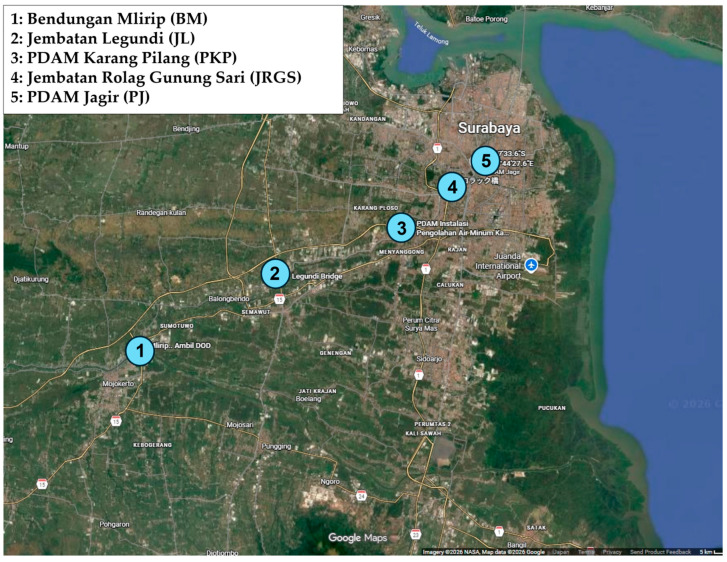
Map of five water sampling sites in the river in Surabaya (downstream site) and province of East Java (upstream site): Bendungan Mlirip (Mlirip dam; upstream site), Jembatan Legundi (Legundi bridge), PDAM Karang Pilang (Karang Pilang regional water company), Jembatan Rolag Gunung Sari (Rolag Bridge Gunung Sari), and PDAM Jagir (Jagar regional water company; downstream site).

**Figure 3:**
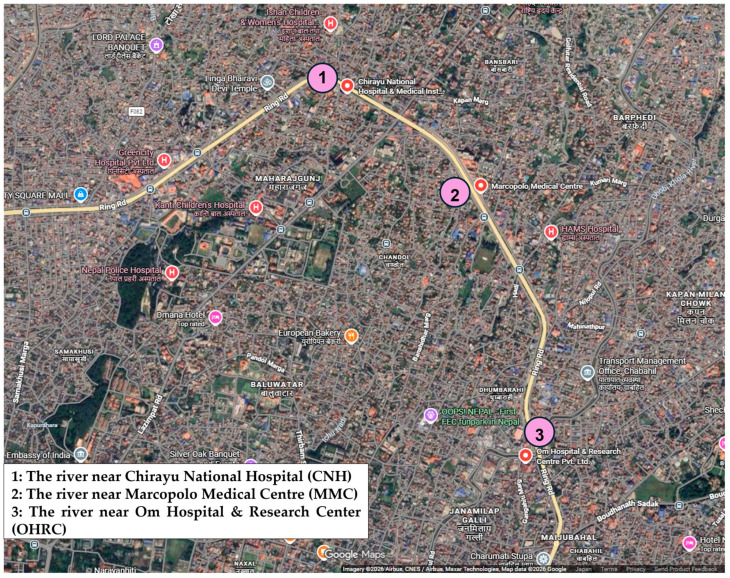
Map of three water sampling sites in the downstream site of rivers in Kathmandu, Nepal. Hospitals are denoted by red map symbols containing the letter “H.”.

In Japan, six samples were collected in May 2024 from three rivers in Hyogo Prefecture [Akashi River: AKR (upstream site: Akashi city, downstream site: Kobe city), Mukogawa River: MUR (upstream site: Takarazuka city, downstream site: Nishinomiya city), and Awaji River: AWR (upstream and downstream sites: Sumoto city in Awaji Island)] (Figure 1). Within the AKR watershed, no hospitals were in the upstream region; however, one hospital was situated in proximity downstream, and an additional five were distributed throughout the lower reaches, including one adjacent to the river (Figure S1A). Similarly, no hospitals were present upstream of the MUR, whereas one facility was located near downstream and another within the downstream vicinity (Figure S1B). Along the AWR, a single hospital was identified in the downstream area (Figure S1C). In Indonesia, five samples were obtained in February 2024 from five river locations in Surabaya (downstream site) and province of East Java (upstream site): Bendungan Mlirip (BM, Mlirip dam; upstream site), Jembatan Legundi (JL, Legundi bridge), PDAM Karang Pilang (PKP, Karang Pilang regional water company), Jembatan Rolag Gunung Sari (JRGS, Rolag Bridge Gunung Sari), and PDAM Jagir (PJ, Jagar regional water company; downstream site) (Figure 2). One hospital was located near BM, with an additional six hospitals distributed throughout the surrounding upstream region (Figure S2A). One hospital was situated in proximity to JL, and three were in the vicinity of PKP (Figure S2B). Furthermore, two hospitals were identified around JRGS, and six were present in the area surrounding PJ (Figure S2C). In Nepal, three samples were collected in September 2024 from downstream river sites in Kathmandu: the river near Chirayu National Hospital (CNH), the river near Marcopolo Medical Centre (MMC), and the river near Om Hospital & Research Center (OHRC) (Figure 3). All three sites were situated near hospitals, and an additional five hospitals were located within the surrounding area.

### 2.2. Culture Condition and DNA Extraction

From each river sample, 50 mL was collected, and 100 μL was inoculated onto CHROMagar ESBL plates (Kanto Chemical Co., Inc., Tokyo, Japan) and incubated overnight at 37 °C. Colonies growing on the CHROMagar ESBL plates were harvested into 1 mL of physiological saline. Genomic DNA was then extracted from the resulting suspension using the NucleoSpin^®^ Microbial DNA Kit (Macherey-Nagel, Düren, Germany). Samples collected in Japan and Indonesia underwent DNA extraction at Kobe Tokiwa University, whereas those obtained from Nepal were processed at Shi-Gan International College of Science & Technology and then stored at Kobe Tokiwa University. These DNA samples were subjected scNGS and resistome analyses at Kobe Institute of Health.

### 2.3. scNGS Analysis

DNA libraries from samples collected in Japan and Indonesia were prepared using the QIAseq FX DNA Library Kit (Qiagen, Hilden, Germany) and sequenced on the Illumina MiSeq platform (Illumina, San Diego, CA, USA) with v3 chemistry (2 × 300 bp paired-end). DNA libraries from Nepal samples were outsourced for library preparation and sequencing to Bioengineering Lab. Co., Ltd. (Sagamihara, Japan) and sequenced using the DNBSEQ platform (MGI Tech) with 2 × 300 bp paired-end reads. Adapter sequences and low-quality reads were filtered from the raw data using fastp v0.23.2 [18] with def from previously reported data, whereas samples from Japan and Nepal were processed in this study. Sequencing reads from the Japanese and Nepalese samples were taxonomically classified using KrakenUniq v.1.0.4 [19]. Taxonomic relative abundances were summarized at the appropriate taxonomic level using R (v4.2.2) with the vegan package, and the resulting tables were exported in TSV format.

### 2.4. Resistome Analysis

For the detection of ARGs, metagenomic assemblies were generated using SPAdes v.3.15 [20] in metagenomic mode. Assembled contigs were screened for ARGs using ResFinder [21] and only hits with ≥95% sequence identity and ≥90% sequence coverage were included in downstream analyses. Sequencing reads were then mapped to all assembled contigs using BWA v.0.7.4, and mapped reads were processed with SAMtools v.1.15. The number of reads mapped to each contig was determined, and contig abundance was quantified by calculating reads per kilobase per million mapped reads (RPKM), normalized by contig length and total mapped reads. RPKM values for ARG-associated contigs were subsequently extracted and used as indicators of ARG abundance.

### 2.5. Statistical Analysis

Kruskal–Wallis and Steel–Dwass tests, as well as non-metric multidimensional scaling (NMDS) and diversity index analyses, were conducted using BellCurve for Excel (Social Survey Research Information Co., Ltd., Tokyo, Japan). Statistical significance was defined as a *p-*value < 0.05.

## 3. Results

### 3.1. Diversity of ARB in Rivers of Japan, Indonesia, and Nepal

**Figure 4:**
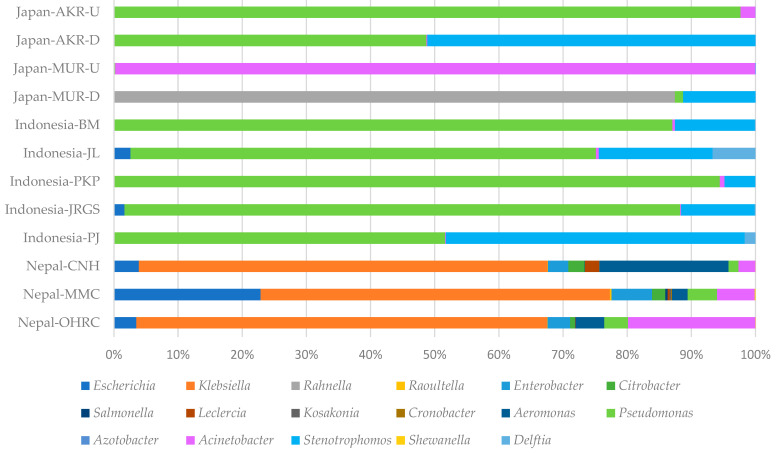
Taxonomic distribution at the genus level of river water culture samples on CHROMagar ESBL plates in Japan, Indonesia and Nepal by metagenomic DNA-Seq analysis.

**Figure 5:**
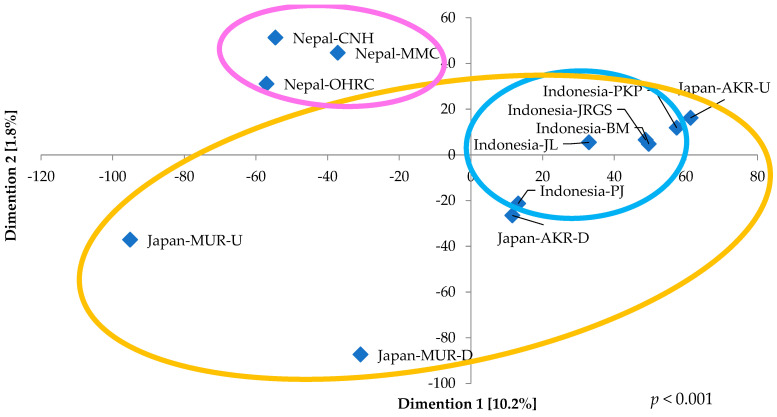
The non-metric multidimensional scaling (NMDS) analysis of genus-level profiles for each river water culture sample from Japan, Indonesia, and Nepal. The yellow circle represents the arrangement of samples in Japan, the blue circle represents it in Indonesia, and the pink circle represents it in Nepal. The *p*-value was derived from the Kruskal–Wallis test comparing the respective groups.

Figure 4 illustrates the taxonomic composition of microbial communities’ culture samples on CHROMagar ESBL plates in river-in systems across Japan, Indonesia, and Nepal. No bacterial colonies were detected on CHROMagar ESBL plates from either the upstream or downstream sites of the AWR in Japan. At the upstream site of the Akashi River (Japan-AKR-U), *Pseudomonas* overwhelmingly dominated the community, accounting for 97.6% of the total relative abundance, whereas the downstream site (Japan-AKR-D) exhibited a near-equal distribution of *Pseudomonas* (48.7%) and *Stenotrophomonas* (51.1%). Similarly, the upstream site of the Mukogawa River (Japan-MUR-U) was characterized by an extreme dominance of *Acinetobacter* (98.7%); however, the downstream site (Japan-MUR-D) was largely composed of *Rahnella* (86.8%) and *Stenotrophomonas* (11.2%), indicating a marked alteration in community composition. In Indonesia, samples collected from five sites along an upstream-to-downstream gradient consistently revealed *Pseudomonas* as the predominant genus, with its highest relative abundance (94.3%) observed at the third sampling site (Indonesia-PKP) and the lowest (51.6%) at the most downstream site (Indonesia-WT). *Stenotrophomonas* represented the second most abundant genus, with relative abundances ranging from 4.8% to 46.6%, reaching its maximum at the upstream Indonesia-WT site. In contrast, downstream sections of three rivers in Nepal were dominated by *Klebsiella*, which comprised approximately 60% of the microbial communities (CNH: 63.7%, MMC: 54.3%, OHRC: 64.0%). The subsequent dominant genera were *Aeromonas* (20.1%) at Nepal-CNH, *Escherichia* (22.8%) at Nepal-MMC, and *Acinetobacter* (19.8%) at Nepal-OHRC. Overall, three to four major bacterial groups were identified across four samples from two Japanese rivers, four to six groups across five Indonesian samples, and fourteen groups across three Nepalese samples, collectively demonstrating substantially higher microbial diversity in the rivers of Nepal. In the NMDS analysis, samples from Nepal and Indonesia demonstrated relatively high intra-regional similarity, as evidenced by their clustered spatial configuration, whereas samples from Japan exhibited comparatively low inter-site similarity (Figure 5). Moreover, statistical assessment using the Kruskal–Wallis test indicated a significant overall difference (*p* < 0.001). Subsequent pairwise comparisons conducted with the Steel–Dwass test revealed significant differences between individual regions in Nepal and Japan-MUR-U or Japan-AKR-U, respectively (Japan-MUR-U vs. Nepal-OHRC, *p* = 0.0364; vs. Nepal-MMC, *p* = 0.0197; vs. Nepal-CNH, *p* = 0.0323 and Japan-AKR-U vs. Nepal-MMC, *p* = 0.049) (Table S1).

### 3.2. Abundance Levels of ARGs in ARB in Japan, Indonesia, and Nepal

**Figure 6:**
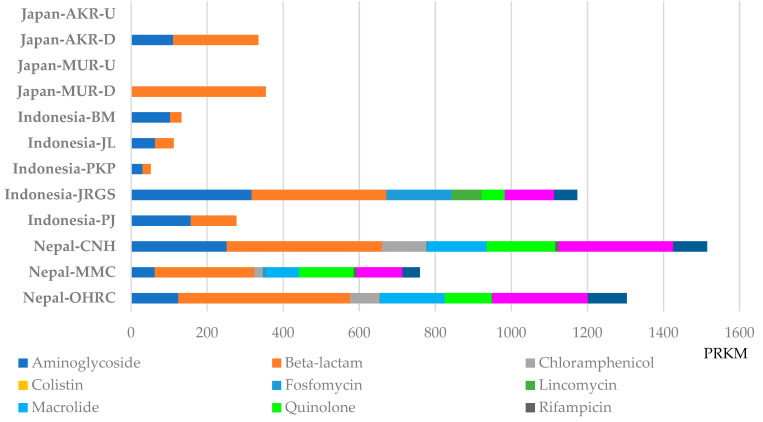
Metagenomic analysis for antimicrobial resistant genes (ARGs) of each culture sample from river water in Japan, Indonesia and Nepal. Metagenomic DNA-seq short reads were analyzed by ResFinder, BWA v.0.7.4 and SAMtools v.1.15, followed by normalization with RPKM. RPKM, per kilobase per million mapped reads.

**Figure 7:**
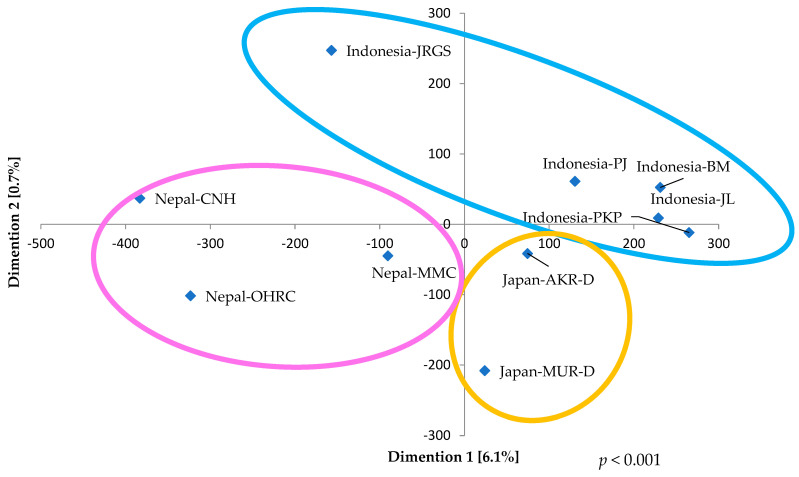
The non-metric multidimensional scaling (NMDS) analysis of multiple comparisons of ARGs among individual river water culture samples from Japan, Indonesia, and Nepal. The yellow circle represents the arrangement of samples in Japan, the blue circle represents it in Indonesia, and the pink circle represents it in Nepal. The *p*-value was derived from the Kruskal–Wallis test comparing the respective groups.

ARGs were identified from metagenomic assemblies, with their abundances quantified as RPKM through read-mapping–based normalization. Each ARG was identified from a single contig per gene within each sample, although multiple distinct ARGs were occasionally located on the same contig. This approach enabled comparative profiling of ARGs in river samples from Japan, Indonesia, and Nepal [11]. Among the identified ARGs classes, resistance determinants associated with aminoglycosides and β-lactams were detected in all three countries (Figure 6, Table S2). The highest abundances of ARGs were recorded in Nepal, reaching 1514.8 RPKM at Nepal-CNH and 1303.7 RPKM at Nepal-OHRC. In these samples, β-lactam resistance genes were predominant (408.6 RPKM at Nepal-CNH and 452.6 RPKM at Nepal-OHRC), followed by sulfamethoxazole/trimethoprim resistance genes (300.3 RPKM at Nepal-CNH and 251.1 RPKM at Nepal-OHRC). The next highest ARG abundance was observed at the downstream site, Indonesia-JRGS (1173.4 RPKM), where a diverse array of ARGs, primarily conferring resistance to β-lactams and aminoglycosides, was detected. In contrast, the remaining Indonesian sites—Indonesia-PJ (277.3 RPKM), Indonesia-PKP (52.0 RPKM), Indonesia-JL (112.0 RPKM), and Indonesia-BM (132.4 RPKM)—exhibited comparatively low ARG levels. At these locations, only aminoglycoside- and β-lactam–resistance genes were identified, with the downstream site, Indonesia-PJ, showing relatively higher abundance than the other non-JRGS sites. In Japan, ARGs were detected exclusively at Japan-AKR-D and Japan-MUR-D. At Japan-MUR-D, only β-lactam resistance genes were observed (354.3 RPKM), whereas at Japan-AKR-D, both β-lactam (224.1 RPKM) and aminoglycoside (110.7 RPKM) resistance genes were present. Based on the NMDS analysis of ARGs, samples from Japan and Nepal were positioned in proximity, indicating high inter-regional similarity, whereas the Indonesian sample (Indonesia-JRGS) exhibited comparatively lower similarity to the other regions (Figure 7). Statistical evaluation using the Kruskal–Wallis test revealed significant overall differences (*p* < 0.001). Subsequent pairwise comparisons conducted with the Steel–Dwass test identified significant differences between Nepal-OHRC and Japan-MUR-U, as well as Indonesia-BM, -JL, and -PKP (Nepal-OHRC Vs. Japan-MUR-U, *p* = 0.0458; Vs. Indonesia-BM, *p* = 0.0488; Vs. Indonesia-JL, *p* = 0.0317; Vs. Indonesia-PKP, *p* = 0.0317) (Table S3).

### 3.3. ARG Types in Riverine Microbiota from Japan, Indonesia, and Nepal

As described above, elevated abundance levels of ARGs were detected in all river samples from Nepal and from the second downstream site, Indonesia-JRGS. In Nepal, the ESBL gene, *bla*CTX-M-15, exhibited the highest abundance levels, reaching 179.4 RPKM at Nepal-CNH, 194.0 RPKM at Nepal-MMC, and 132.7 RPKM at Nepal-OHRC (Table 1; Table S2). This was followed by the sulfonamide resistance gene (*sul1*), with abundance levels of 187.7 RPKM at Nepal-CNH and 122.5 RPKM at Nepal-OHRC; the macrolide resistance gene (*mph(A)*), which showed 126.7 RPKM at Nepal-CNH and 145.5 RPKM at Nepal-OHRC; and the quinolone resistance gene (*qnrS1*), which exhibited high abundance levels of 128.4 RPKM at Nepal-CNH, 132.5 RPKM at Nepal-MMC, and 101.1 RPKM at Nepal-OHRC. Although expressed at comparatively lower levels, carbapenemase genes (*bla*NDM-5, *bla*OXA-181, and *bla*OXA-484) were also detected in Nepal, along with the colistin resistance gene *mcr-1.26*, which was identified exclusively at Nepal-OHRC. In contrast, at Indonesia-JRGS, the ESBL gene (*bla*CTX-M-55) and penicillinase (*bla*TEM-1) were the most abundantly expressed ARGs, with abundance levels of 172.1 RPKM and 135.4 RPKM, respectively.

## 4. Discussion

In this study, in addition to Japan, we analyzed samples collected from multiple riverine environments, including upstream and downstream sites, across different regions such as Indonesia and Nepal, to investigate the diversity of ARB and the distribution of ARGs in river systems. The samples were cultured on CHROMagar ESBL medium, and the obtained ARB are limited to growth on that ESBL medium. In Hyogo Prefecture, located in western Japan, bacterial communities were examined in both the upper and lower reaches of three rivers. Notably, two samples obtained from the upstream and downstream sections of the Awaji River exhibited no bacterial growth on the medium, and no ARBs were detected. This finding suggests that the dissemination of ARB is geographically limited on the Awaji River in Awaji Island, in southern Hyogo, likely due to its geographic isolation and distance from major urban centers. In contrast, in the Akashi River in western Hyogo, *Pseudomonas* and *Stenotrophomonas* were detected, whereas in the Mukogawa River in eastern Hyogo, *Acinetobacter* was identified at upstream sites and *Rahnella* at downstream sites. In Japan, the similarity of bacterial community composition varied among rivers; however, no statistically significant differences were observed between rivers. In contrast, significant differences in community similarity were detected when compared with bacterial communities in Nepalese rivers (all *p* < 0.05; Table S1).

Furthermore, no ARGs were detected at upstream sites of the Akashi and Mukogawa Rivers. In the downstream regions, likely influenced by the proximity of hospitals (Figure S1), β-lactam resistance genes were identified; nevertheless, their relative abundance remained low. This observation is consistent with antibiotic usage patterns in Japan, where β-lactam antibiotics predominate [22]. A previous investigation of the Yodo River basin in Osaka—one of the major river systems in western Japan and located next to Hyogo Prefecture—reported the presence of carbapenemase and ESBL genes in river water samples, indicating incomplete removal of these ARGs during wastewater treatment [13]. Although the Mukogawa River is geographically close to the Yodo River, it is considerably less urbanized, which likely explains the absence of detectable ARGs in this study. In eastern Japan, aminoglycoside and tetracycline resistance genes have been detected in the Kanda River in Tokyo, whereas studies of water sources in the mountainous region of Yamanashi reported low detection frequencies of ARGs, including integrase gene and *sul1*, with commonly observed urban ESBL-associated genes being absent [14,15]. The downstream regions of the Akashi and Mukogawa Rivers are less urbanized than the Kanda River and, unlike Yamanashi, are not predominantly mountainous and have limited livestock farming and fewer hospitals. Consequently, the observed results appear to fall between those reported for the Kanda River and the Yamanashi region. In Japan, although ESBL-producing strains are frequently detected [7], carbapenem-resistant strains are comparatively rare [8,23], and it is plausible that the findings of the riverine survey reflect this epidemiological pattern.

Previous analysis of six urban river samples from Indonesia revealed that the genera *Acinetobacter* and *Pseudomonas* were detected at all sampling sites. However, near hospital-adjacent locations, the relative abundance of these genera was lower, while members of the order *Enterobacterales* were more prevalent [11]. Another survey of water collection points along the Code and Merapi Rivers in Indonesia demonstrated that *Burkholderiales* and *Rhizobiales* dominated spring water sources, whereas fecal-associated gut bacteria were detected at rural collection points near cattle farms, reflecting agricultural influences [24]. In the present study, river water samples were collected from five sites spanning from upstream headwaters to urban downstream areas in Indonesia. *Pseudomonas* was identified as the dominant genus at four of the five sites, followed by *Stenotrophomonas.* Although *Pseudomonas* was consistently detected, as reported previously, the relatively low abundance of *Enterobacterales* is likely attributable to the absence of nearby sewage inflows at the sampling sites.

With respect to ARG distribution, aminoglycoside and β-lactam resistance genes were predominant in the earlier Indonesian study, with exceptionally high total ARG abundance levels—exceeding approximately 4000 RPKM—at two sites near hospitals [11]. In contrast, in the present study, only the second downstream site in Indonesia (Indonesia-JRGS) exhibited a relatively elevated total ARG abundance (1173.4 RPKM) compared with other locations. Similarly, in the NMDS analysis, the Indonesia-JRGS sample was positioned at a greater distance from the other regions, indicating lower similarity and demonstrating a significant difference (*p* < 0.001; Figure 7). Nevertheless, the magnitude of this difference was substantially lower than previously reported values. Although numerous hospitals are in proximity to each site (Figure S2), these findings may suggest that such proximity has not resulted in substantial contamination of the rivers with ARGs.

In Nepal, water samples were collected from three sites in the urban downstream area of Kathmandu. As all sampling locations were near hospitals, the ARB were dominated by the enteric genus *Klebsiella*, with *Aeromonas*, *Escherichia* and *Acinetobacter*. Among the three countries surveyed, Nepal exhibited the highest bacterial diversity, with particularly significant differences observed in comparison to multiple regions in Japan (all *p* < 0.05; Table S1). Nepal also demonstrated the greatest overall abundance of ARGs, with β-lactam resistance determinants—ESBL genes such as *bla*CTX-M-15—being the most frequently detected. Moreover, carbapenemase genes and the colistin resistance gene were detected. The detection of ARGs conferring resistance to last-line antimicrobials, including carbapenems and colistin, indicates the ongoing dissemination of ARB within hospital settings and urban environments. These resistance determinants are readily transmissible across diverse bacterial taxa through mobile genetic elements, such as plasmids, thereby facilitating their rapid propagation within microbial communities and posing a substantial threat to public health [25].

Previous analyses of river and sewage samples in Nepal in 2016 reported that *sul1* accounted for approximately 94% of detected ARGs, with additional detection of the integrase gene and the tetracycline resistance gene [16]. However, more recent samples collected in 2023 revealed the presence of carbapenemase and ESBL genes in *Enterobacterales*. In parallel, clinical surveys of human and poultry isolates in Nepal identified ESBL-producing *E. coli* with colistin-resistant gene and carbapenemase-producing *P. aeruginosa* [17]. With respect to the detection of drug-resistant bacteria in Nepalese hospitals, data from 2020 to 2021 indicate that 31.2% of 77 isolated *Escherichia coli* strains were carbapenem-resistant, of which 45.9% exhibited resistance to colistin. Furthermore, among 36 *Pseudomonas aeruginosa* isolates, 50% were multidrug-resistant, with 66.7% producing metallo-β-lactamases and 11.2% demonstrating resistance to colistin. These findings suggest that such resistant strains are widely disseminated among clinical isolates, and their detection in riverine environments clearly reflects the regional distribution of ARGs [26,27].

In other Asian countries, surveys of rivers in Sri Lanka and India detected widespread resistance to β-lactams, fluoroquinolones, and sulfonamides [28]. The *qnrS* and *sul1* were identified in rivers in both countries, whereas ESBL genes were characteristic of Indian rivers, which also exhibited a higher overall diversity of ARGs compared with Sri Lanka. In Vietnam, riverine concentrations of β-lactams and sulfonamides have been reported to be higher than those observed in many other regions worldwide [29]. Beyond aquatic environments, another study detected a wide array of ARGs in livestock (e.g., chickens and pigs) and healthy humans, including ESBL gene (*bla*SHV-1), *qnrS*, macrolide resistance genes, and aminoglycoside resistance genes [30]. Similarly, investigations of antimicrobial resistance in non-typhoidal *Salmonella enterica* from chicken meat in Indonesia identified β-lactam, quinolone, and tetracycline resistance genes, confirming that AMR is widespread not only in water environments but also in livestock [10]. Collectively, these findings indicate that ARGs originating from humans and poultry have disseminated into river systems, and that a potential cycle of ARG transmission from rivers back to humans and animals via drinking water may have been established. Consequently, the increasing detection of ESBL genes, carbapenemase genes, and colistin-resistance genes in aquatic environments across multiple countries represents a growing concern for clinical practice.

Global consumption of ARGs increased by 16.3% between 2016 and 2023 [31], with particularly pronounced increases in emerging economies rather than in Asian countries. This trend is consistent with the ARG profiles observed in Indonesia and Nepal in the present study. Resistome analysis revealed that river waters frequently harbor resistance genes against antibiotics commonly used in clinical settings. Notably, Nepal exhibited both high bacterial diversity and high total ARG abundance levels. In addition to previous findings from Indonesia, clinically significant resistance determinants, including ESBLs, as well as high-risk genes such as *bla*NDM and *mcr*, were detected in rivers near hospitals in urban areas of Nepal, highlighting the existence of multiple reservoirs and transmission pathways for ARGs. While countries such as Japan have implemented stringent measures to promote the appropriate use of antibiotics in medical institutions, comparable regulatory systems are not yet fully established in many emerging countries. Future investigations incorporating diverse sample types in emerging regions, together with the dissemination of findings through collaborative networks, will be essential for establishing evidence-based strategies to support the prudent use of antibiotics.

A limitation of this study is that genomic DNA was extracted exclusively from bacteria cultured on CHROMagar ESBL medium, thereby confining the analysis to selectively enriched bacterial populations. Consequently, the dataset does not encompass the full microbial community present in the water samples and should be interpreted as a genomic analysis of culture-enriched bacteria. Accordingly, any inferences regarding overall riverine microbial diversity and the distribution of ARGs are restricted to bacterial taxa that are both culturable and selectively enriched under ESBL-targeted conditions. Moreover, because DNA was not isolated from individual colonies, strain-level comparisons and sequence type analyses could not be performed. Additionally, the limited number of samples obtained at each site, together with the inability to collect upstream samples in Nepal, suggests that the findings are likely influenced by hospital wastewater discharge. Furthermore, as the sampling design was not rigorously standardized across the three countries, the validity of direct cross-national comparisons is inherently constrained.

## 5. Conclusions

In conclusion, advanced scNGS and resistome analyses of environmental water samples derived from CHROMagar ESBL media revealed pronounced spatial variations in ARB from upstream to downstream sites in Japan, Indonesia, and Nepal, alongside a notable prevalence of ARGs, particularly those linked to the genus *Klebsiella* in urban downstream regions of Nepal. Cross-country comparisons further elucidated the extent and patterns of riverine contamination by diverse ESBL-related ARGs. Future research should integrate comparative analyses of ARG profiles derived from human and poultry isolates with those detected in river samples to enable a more comprehensive assessment of the transmission and circulation of ARGs among aquatic environments, humans, and poultry.

## Figures and Tables

**Table 1:** Genotype of antimicrobial resistant gene (ARG) type of river water samples in Japan, Indonesia and Nepal.

Sample *	ARG Type
**Japan-AKR-D**	*aph(6)-Smalt*, *bla*L1, *bla*L2
**Japan-MUR-D**	*bla*L1, *bla*L2, *bla*RAHN-2
**Indonesia-BM**	*aac(6′)-Iz*, *aph(3′)-Iic*, *aph(6)-Smalt*, *bla*L1
**Indonesia-JL**	*aph(3′)-Iic*, *aph(6)-Smalt*, *bla*CTX-M-15, *bla*EC-5, *bla*L2
**Indonesia-PKP**	*aph(3′)-Iic*, *bla*L1, *bla*L2
**Indonesia-JRGS**	*aac(3)-Iid*, *aac(6′)-Iak*, *aadA2*, *aph(3′)-Iic*, *aph(3″)-Ib*, *aph(6)-Id*
	*aph(6)-Smalt*, *bla*CTX-M-55, *bla*EC, *bla*TEM-1, *fosA3*, *fosA4*,
	*lnu(F)*, *qnrS13*, *sul2*, *dfrA14*, *tet(A)*
**Indonesia-PJ**	*aac(6′)-Iz*, *aph(3′)-Iic*, *aph(6)-Smalt*, *blaL1*, *blaL2*,
**Nepal-CNH**	*aac(3)-IIa*, *aac(3)-IV*, *aac(6′)-Ib-cr*, *aac(6′)-Ib-cr*, *aac(6′)-Il*, *aadA1*, *aadA16*, *aadA2*, *aadA5*, *ant(2″)-Ia*, *aph(3″)-Ib*, *aph(3′)-Ia*, *aph(3′)-IIb*, *aph(3′)-VI*, *aph(4)-Ia*, *ARR-6*, *bla*ACT-15, *bla*CMY-42, *bla*CTX-M-15, *bla*CTX-M-27, *bla*DHA-1, *bla*KPC-2, *bla*LAP-2, *bla*NDM-5, *bla*OXA-1, *bla*OXA-10, *bla*OXA-2, *bla*OXA-484, *bla*OXA-50, *bla*OXA-88, *bla*PER-3, *bla*SHV-148, *bla*TEM-234, *bla*VEB-1, *catA1*, *catB3*, *catB8*, *cmlA1*, *crpP*, *dfrA1*, *dfrA12*, *dfrA14*, *dfrA17*, *dfrA27*, *dfrA5*, *erm(B)*, *floR*, *fosA*, *mph(A)*, *mph(E)*, *msr(E)*, *OqxA*, *OqxB*, *qepA4*, *qnrB1*, *qnrB4*, *qnrS1*, *qnrS2*, *qnrVC1*, *qnrVC4*, *rmtB*, *rmtF*, *sul1*, *sul2*, *sul3*, *tet(39)*, *tet(A)*, *tet(B)*, *tet(C)*, *tet(E)*
**Nepal-MMC**	*aac(3)-IId*, *aac(6′)-Ib-cr*, *aac(6′)-Ib-cr*, *aadA2*, *aadA5*, *aph(3″)-Ib*, *aph(3′)-Ia*, *aph(6)-Id*, *ARR-2*, *bla*ACT-16, *bla*CMY-42, *bla*CTX-M-15, *bla*CTX-M-27, *bla*DHA-1, *bla*NDM-5, *bla*OXA-1, *bla*OXA-181, *bla*OXA-98, *bla*PER-3, *bla*SHV-81, *bla*TEM-234, *catB3*, *cmlA1*, *dfrA1*, *dfrA12*, *dfrA14*, *dfrA15*, *erm(B)*, *fosA6*, *mph(A)*, *OqxA*, *OqxB*, *qnrB1*, *qnrB4*, *qnrS1*, *qnrVC1*, *sul1*, *sul2*, *tet(A)*, *tet(B)*
**Nepal-OHRC**	*aac(3)-IId*, *aac(3)-IV*, *aac(6′)-Ib-cr*, *aac(6′)-Ib-cr*, *aac(6′)-Il*, *aadA1*, *aadA16*, *aadA2*, *aadA5*, *ant(2″)-Ia*, *aph(3″)-Ib*, *aph(3′)-Ia*, *aph(4)-Ia*, *ARR-6*, *bla*ACT-16, *bla*CMY-145, *bla*CTX-M-15, *bla*CTX-M-27, *bla*DHA-1, *bla*KPC-2, *bla*LAP-2, *bla*NDM-5, *bla*OXA-1, *bla*OXA-181, *bla*OXA-50, *bla*OXA-64, *bla*PER-3, *bla*SFO-1, *bla*SHV-80, *bla*TEM-1B, *catA1*, *catB3*, *cmlA1*, *dfrA1*, *dfrA12*, *dfrA14*, *dfrA17*, *dfrA5*, *erm(B)*, *floR*, *fosA*, *mcr-1.26*, *mph(A)*, *mph(E)*, *msr(E)*, *OqxA*, *OqxB*, *POM-1*, *qepA4*, *qnrB1*, *qnrB4*, *qnrS1*, *rmtB*, *rmtF*, *sul1*, *sul2*, *sul3*, *tet(39)*, *tet(A)*, *tet(B)*, *tet(C)*, *tet(E)*, *tet(X4)*

* Japan: The downstream site of Akashi River (AKR-D), The downstream site of Mukogawa River (MUR-D); Indonesia: Bendungan Mlirip (BM), Jembatan Legundi (JL), PDAM Karang Pilang (PKP), Jembatan Rolag Gunung Sari (JRGS), PDAM Jagir (PJ); Nepal: The river near Chirayu National Hospital (CNH), the river near Marcopolo Medical Centre (MMC), and the river near Om Hospital & Research Center (OHRC).

## Data Availability

The raw sequencing data generated in this study have been deposited in the DDBJ/NCBI/EMBL databases under accession numbers DRR911260–DRR911274.

## Supplementary Materials

The following supporting information can be downloaded at: https://www.mdpi.com/article/10.3390/pathogens15030317/s1, Figure S1. A detailed geographic representation of six water sampling sites distributed across three river systems in Hyogo Prefecture, Japan. Figure S2. A detailed geographic representation of five water sampling sites distributed across river systems in Surabaya, Indonesia. Table S1: Statistical analysis of multiple comparisons of the genus level among individual river water culture samples from Japan, Indonesia, and Nepal. Table S2. Profiling of antimicrobial resistant genes (ARGs) of river water samples in Japan, Indonesia and Nepal. Table S3. Statistical analysis of multiple comparisons of ARGs among individual river water culture samples from Japan, Indonesia, and Nepal.

## Abbreviations

The following abbreviations are used in this manuscript:

AMR	antimicrobial resistance
ARB	antimicrobial-resistant bacteria
ARGs	antimicrobial resistant genes
AKR	Akashi River (Japan)
AKR-D	The downstream site of Akashi River
AKR-U	The upstream site of Akashi River
AWR	Awaji River (Japan)
AWR-D	The downstream site of Awaji River
AWR-U	The upstream site of Awaji River
BM	Bendungan mlirip (Indonesia)
CARD	Comprehensive antibiotic resistance database
CNH	Chirayu National Hospital (Nepal)
ESBL	extended-spectrum β-lactamase
JL	Jembatan Legundi (Indonesia)
JRGS	Jembatan Rolag Gunung Sari (Indonesia)
MMC	Marcopolo Medical Centre (Nepal)
MUR	Mukogawa River (Japan)
MUR-D	The downstream site of Mukogawa River
MUR-U	The upstream site of Mukogawa River
NMDS	Non-metric multidimensional scaling
OHRC	Om Hospital & Research Center (Nepal)
OTUs	operational taxonomic units
PJ	PDAM jagir (Indonasia)
PKP	PDAM karang pilang (Indonesia)
RGI	resistance gene identifier
RPKM	reads per kilobase per million mapped read
scNGS	selective culture–based Next Generation Sequencing
