# Supplementary material for: Survey of Antimicrobial-Resistant Bacteria Isolated from Rivers in Japan, Indonesia and Nepal
Source: Pathogens. 2026 Mar 15;15(3):317. doi: 10.3390/pathogens15030317 (PMC13028957; doi:10.3390/pathogens15030317)
Supplement: Supplementary file 1 [file pathogens-15-00317-s001.zip › pathogens-4151911-supplementary.pdf]

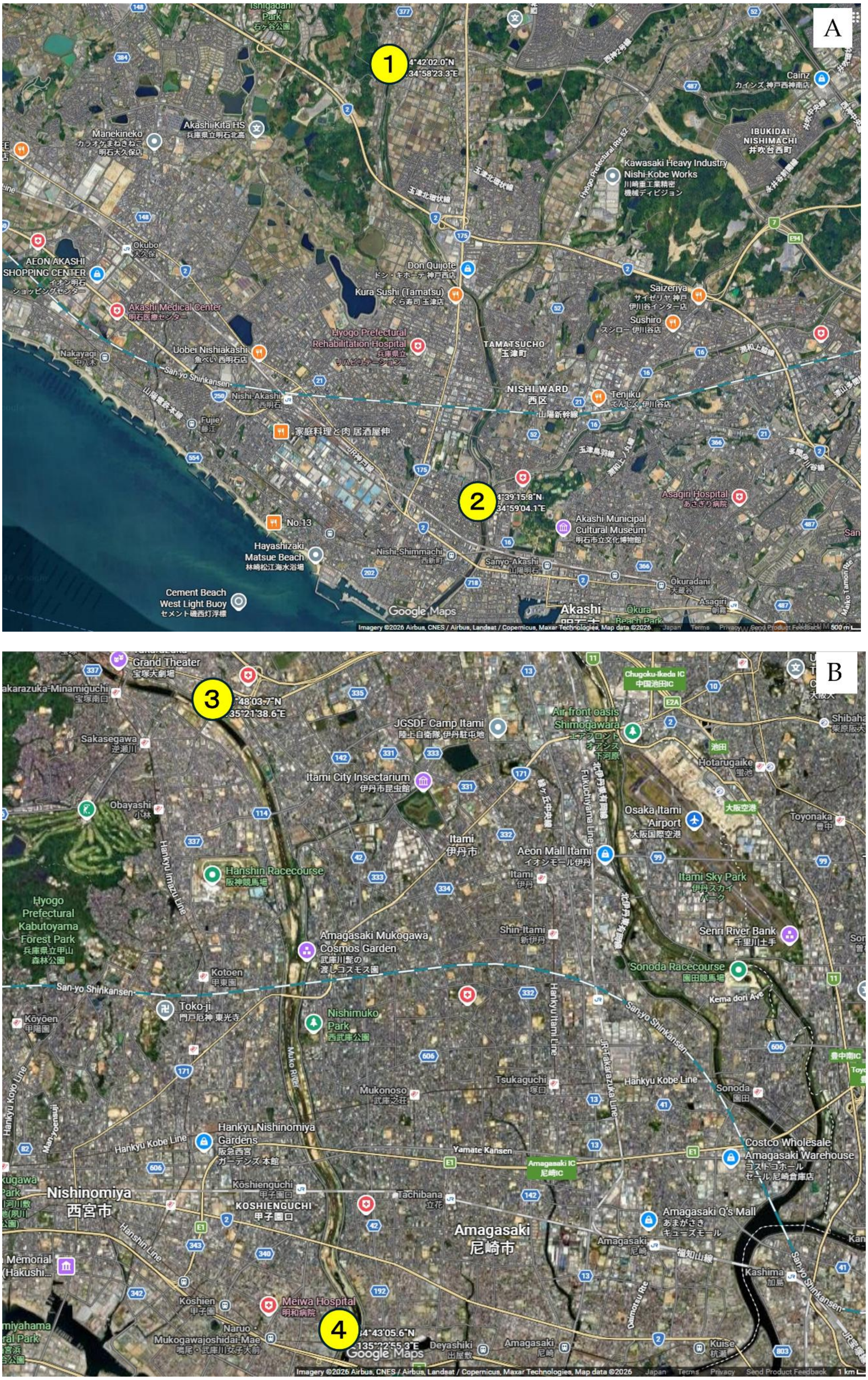

Figure S1. A detailed geographic representation of six water sampling sites distributed across three river systems in Hyogo Prefecture, Japan. A: 1, The upstream site of Akashi river (AKR-U) and 2, The downstream site of Akashi river (AKR-D); B: 3, The upstream site of Mukogawa river (MUR-U) and 4, The downstream site of Mukogawa river (MUR-D); C: 5, The upstream site of Awaji river (AWR-U) and 6, The downstream site of Awaji river (AWR-D), The hospital map symbol is red with a red cross inside.

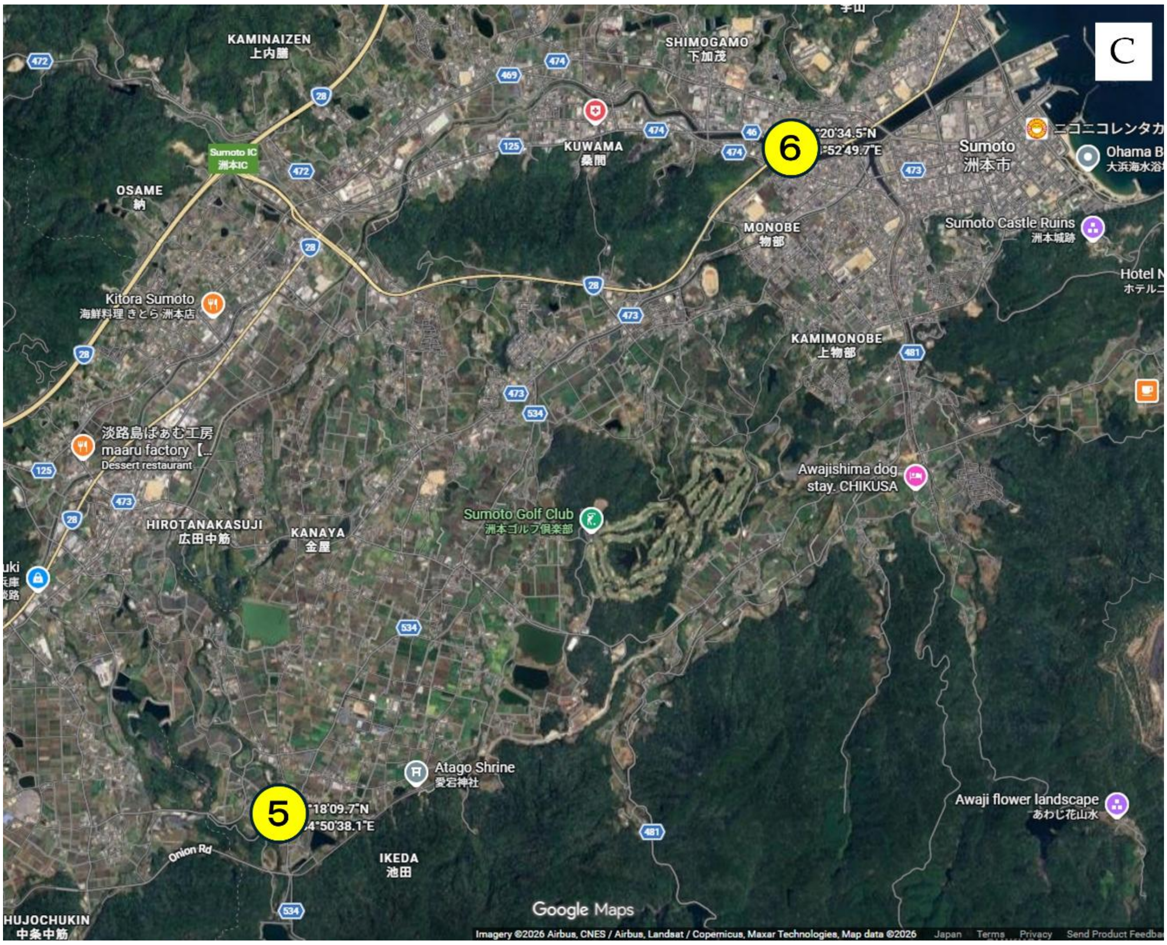

Figure S1. continued.

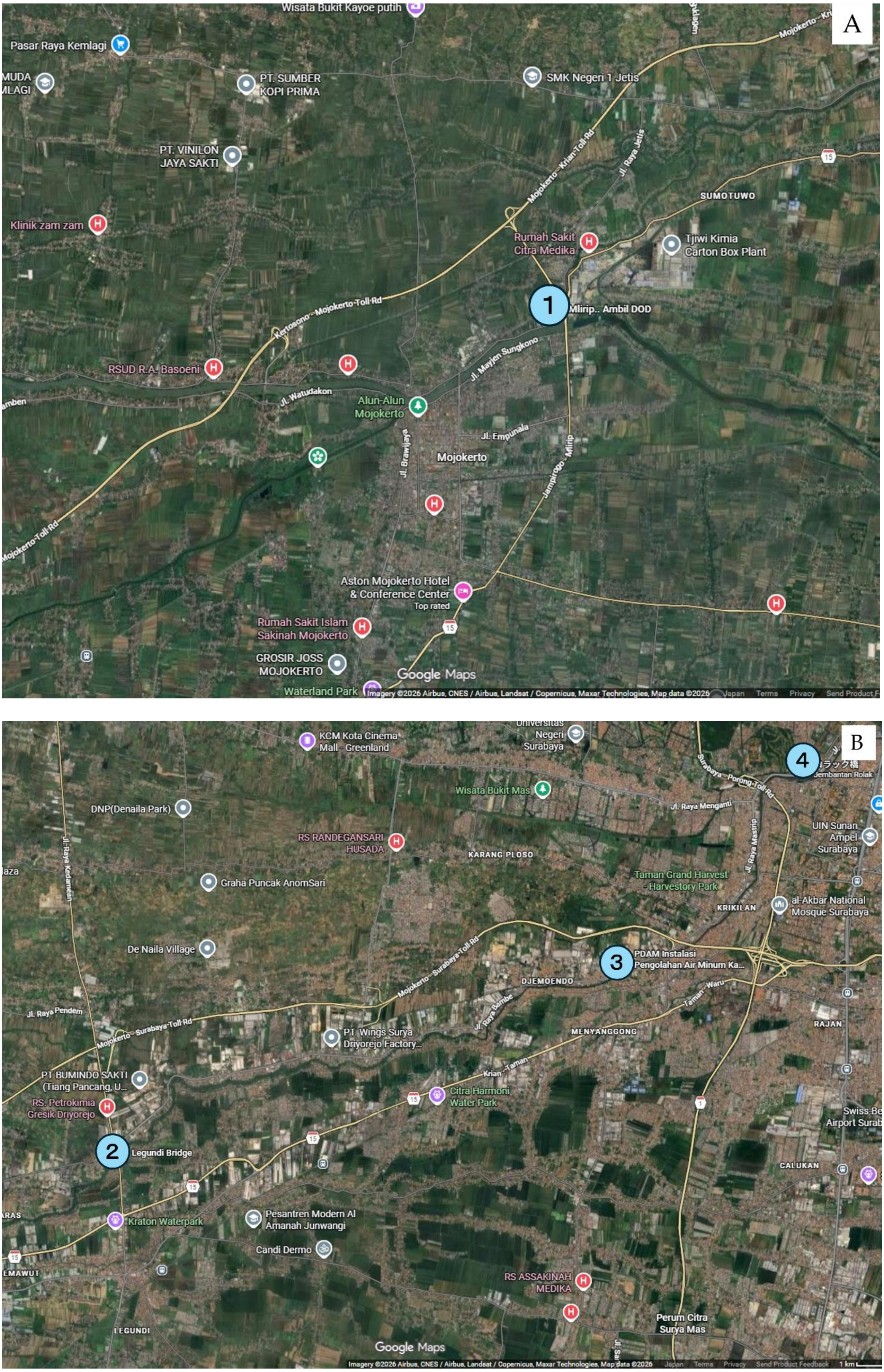

Figure S2. A detailed geographic representation of five water sampling sites distributed across river systems in Surabaya, Indonesia. A: 1, Bendungan Mlirip (BM); B: 2, Jembatan Legundi (JL), 3, PDAM Karang Pilang (PKP) and 4, Jembatan Rolag Gunung Sari (JRGS); C: 4, JRGS and 5, PDAM Jagir (PJ). The hospital map symbol is red with "H" inside.

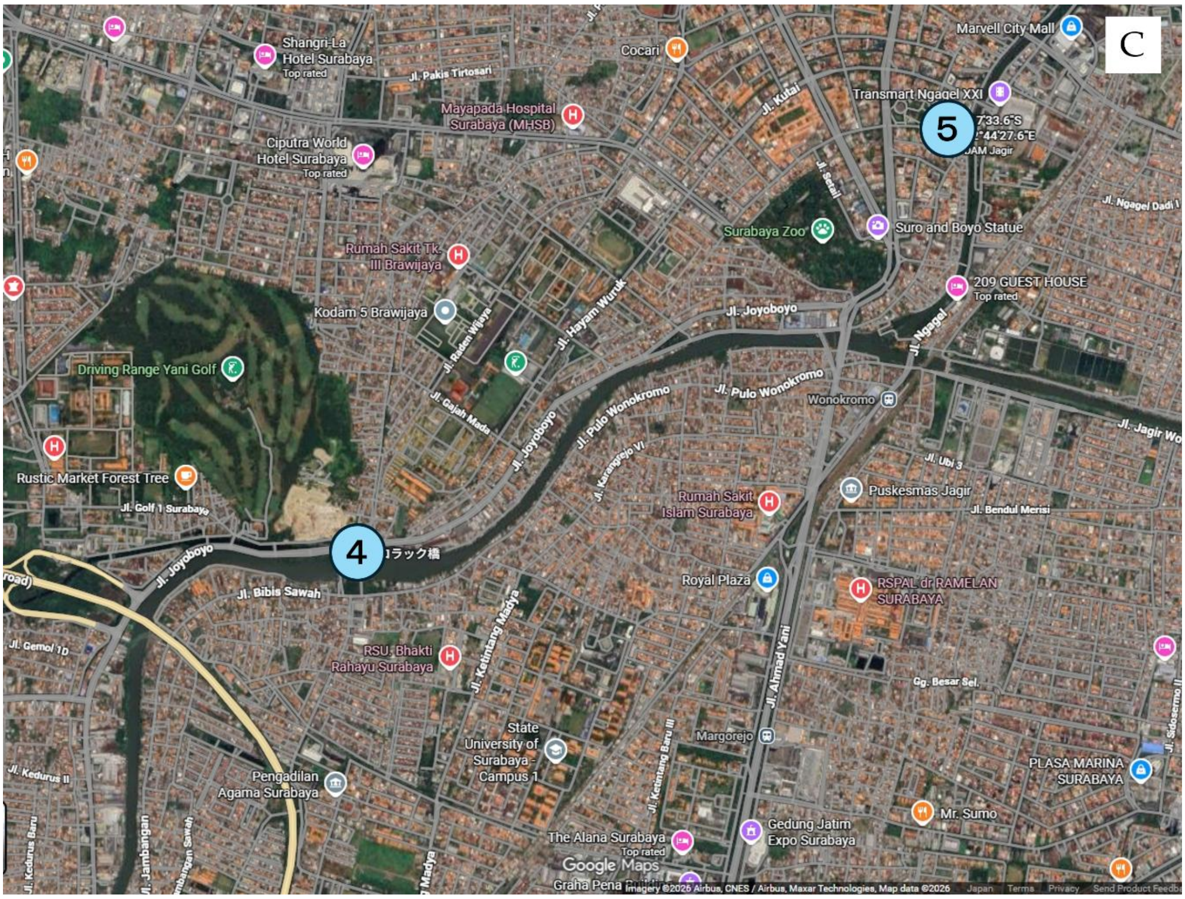

Figure S2. Continued.

Table S1. Statistical analysis of multiple comparisons at the genus level in river water culture samples on CHROMagar ESBL media from Japan, Indonesia, and Nepal

Level 1	Level 2	*p* -value
Nepal-OHRC	Nepal-MMC	0.9996
Nepal-OHRC	Nepal-CNH	1.0000
Nepal-OHRC	Indonesia-PJ	0.3152
Nepal-OHRC	Indonesia-JRGS	0.4857
Nepal-OHRC	Indonesia-PKP	0.2963
Nepal-OHRC	Indonesia-JL	0.6177
Nepal-OHRC	Indonesia-BM	0.3376
Nepal-OHRC	Japan-MUR-D	0.3399
Nepal-OHRC	Japan-MUR-U	0.0364 *
Nepal-OHRC	Japan-AKR-D	0.1369
Nepal-OHRC	Japan-AKR-U	0.0681
Nepal-MMC	Nepal-CNH	1.0000
Nepal-MMC	Indonesia-PJ	0.1994
Nepal-MMC	Indonesia-JRGS	0.2532
Nepal-MMC	Indonesia-PKP	0.1862
Nepal-MMC	Indonesia-JL	0.6437
Nepal-MMC	Indonesia-BM	0.1834
Nepal-MMC	Japan-MUR-D	0.2021
Nepal-MMC	Japan-MUR-U	0.0197 *
Nepal-MMC	Japan-AKR-D	0.0929
Nepal-MMC	Japan-AKR-U	0.0490 *
Nepal-CNH	Indonesia-PJ	0.2937
Nepal-CNH	Indonesia-JRGS	0.4091
Nepal-CNH	Indonesia-PKP	0.2559
Nepal-CNH	Indonesia-JL	0.6177
Nepal-CNH	Indonesia-BM	0.2730
Nepal-CNH	Japan-MUR-D	0.2370
Nepal-CNH	Japan-MUR-U	0.0323 *
Nepal-CNH	Japan-AKR-D	0.1369
Nepal-CNH	Japan-AKR-U	0.0611
Indonesia-PJ	Indonesia-JRGS	1.0000
Indonesia-PJ	Indonesia-PKP	1.0000
Indonesia-PJ	Indonesia-JL	1.0000
Indonesia-PJ	Indonesia-BM	1.0000
Indonesia-PJ	Japan-MUR-D	1.0000
Indonesia-PJ	Japan-MUR-U	0.9998

Indonesia-PJ	Japan-AKR-D	1.0000
Indonesia-PJ	Japan-AKR-U	1.0000
Indonesia-JRGS	Indonesia-PKP	1.0000
Indonesia-JRGS	Indonesia-JL	1.0000
Indonesia-JRGS	Indonesia-BM	1.0000
Indonesia-JRGS	Japan-MUR-D	1.0000
Indonesia-JRGS	Japan-MUR-U	0.9997
Indonesia-JRGS	Japan-AKR-D	1.0000
Indonesia-JRGS	Japan-AKR-U	1.0000
Indonesia-PKP	Indonesia-JL	1.0000
Indonesia-PKP	Indonesia-BM	1.0000
Indonesia-PKP	Japan-MUR-D	1.0000
Indonesia-PKP	Japan-MUR-U	0.9967
Indonesia-PKP	Japan-AKR-D	1.0000
Indonesia-PKP	Japan-AKR-U	1.0000
Indonesia-JL	Indonesia-BM	1.0000
Indonesia-JL	Japan-MUR-D	1.0000
Indonesia-JL	Japan-MUR-U	0.9991
Indonesia-JL	Japan-AKR-D	1.0000
Indonesia-JL	Japan-AKR-U	1.0000
Indonesia-BM	Japan-MUR-D	1.0000
Indonesia-BM	Japan-MUR-U	0.9998
Indonesia-BM	Japan-AKR-D	1.0000
Indonesia-BM	Japan-AKR-U	1.0000
Japan-MUR-D	Japan-MUR-U	0.9978
Japan-MUR-D	Japan-AKR-D	0.9999
Japan-MUR-D	Japan-AKR-U	1.0000
Japan-MUR-U	Japan-AKR-D	1.0000
Japan-MUR-U	Japan-AKR-U	1.0000
Japan-AKR-D	Japan-AKR-U	1.0000

Statistical analysis of multiple comparisons: Steel-Dwass analysis

*：$p<0.05$ **：$p<0.01$

Table S2. Profiling of antimicrobial resistant genes (ARGs) of river water samples in Japan, Indonesia and Nepal.

ARGs		Total	RPKM[#]									
			Japan*		Indonesia*					Nepal*		
			AKR-D	MUR-D	BM	JL	PKP	JRGS	PJ	CNH	MMC	OHRC
**Aminoglycoside**	**overall**	1218.7	110.7	0.0	102.9	63.3	30.4	316.8	156.8	251.5	62.1	124.2
	aac(3)-Iia	25.7								25.7		
	aac(3)-Iid	105.4						88.8				16.6
	aac(3)-Iva	10.6								0.9		9.7
	aac(6')-Iak	8.2						8.2				
	aac(6')-Ib-cr	85.0								57.4	10.3	17.2
	aac(6')-Il	4.7								3.6		1.1
	aac(6')-Iz	55.5			37.4				18.2			
	aadA1	29.6								11.3		18.3
	aadA2	168.1						78.7		25.9	21.5	41.9
	aadA5	30.1								27.8	2.3	
	aadA6	0.9									0.9	
	aadA16	12.4								10.8		1.6
	ant(2'')-Ia	11.7								10.9		0.9
	aph(3')-Ia	13.5								5.0	1.3	7.2
	aph(3')-IIc	170.5			33.5	34.2	30.4	4.6	67.8			
	aph(3'')-Ib	145.5						64.0		68.7	12.9	
	aph(4)-Ia	10.6								0.9		9.7
	aph(6)-Id	76.9						64.0			12.9	
	aph(6)-Smalt	251.1	110.7		32.0	29.1		8.6	70.8			
	rmtF	2.5								2.5		
**Beta-lactam**	**overall**	2276.9	224.1	354.4	29.5	48.7	21.6	354.9	120.5	408.6	262.1	452.6
	bla ACT-2	0.8									0.8	
	bla ACT-15	3.2								3.2		
	bla ACT-16	5.2									1.3	3.8
	bla ADC-25	7.7								4.4	3.3	
	bla CMY-42	61.5								47.6	13.9	
	bla CMY-145	57.2										57.2
	bla CTX-M-15	519.2				13.1				179.4	194.0	132.7
	bla CTX-M-27	10.0								3.3	2.3	4.4
	bla CTX-M-55	172.1						172.1				
	bla DHA-1	31.3								16.8	2.7	11.7
	bla EC	47.4						47.4				
	bla EC-5	13.4				13.4						
	bla KPC-2	8.9								7.0		1.8
	bla NDM-5	75.6								34.9	5.8	34.8
	bla L1	363.5	110.7	143.0	29.5		17.1		63.2			
	bla L2	353.4	113.4	156.0		22.2	4.5		57.3			
	bla MOX-3	6.8								6.8		
	bla MOX-6	14.8								14.8		
	bla OXA-1	38.5								18.7	10.3	9.4
	bla OXA-2	3.5								3.5		
	bla OXA-10	2.7								2.7		
	bla OXA-50	0.4										0.4
	bla OXA-88	4.3								4.3		
	bla OXA-98	2.2									2.2	
	bla OXA-180	28.1										28.1
	bla OXA-181	36.4									10.7	25.7
	bla OXA-396	55.4		55.4								
	bla OXA-484	12.4								12.4		
	bla OXA-506	5.1										5.1
	bla PER-3	15.4								6.8	7.1	1.5
	bla RAHN	0.0										
	bla SHV-110	7.7									7.7	
	bla SHV-187	72.5								36.4		36.0
	bla VEB-1	5.3								5.3		
	bla TEM-1	135.4						135.4				
	bla TEM-1B	97.0										97.0
	POM-1	2.8										2.8
**Chloramphenicol**	**overall**	213.5	0.0	0.0	0.0	0.0	0.0	0.0	0.0	116.3	22.2	75.0
	catA1	23.8								5.8		18.1
	catB8	2.4								2.4		
	CmlA7	10.1								6.7	1.7	1.8
	FloR	28.8								16.7		12.1
	OqxA	95.8								42.4	10.3	43.1
	OqxB	52.6								42.4	10.3	
**Colistin**	*mcr-1*	1.2										1.2
**Fosfomycin**	**overall**	190.4	0.0	0.0	0.0	0.0	0.0	171.4	0.0	5.6	10.7	2.8
	fosA	8.4								5.6		2.8
	fosA3	86.5						86.5				
	fosA4	84.8						84.8				
	fosA6	9.9									9.9	
	fosA7	0.8									0.8	
**Lincomycin**	*lnu(F)*	78.7						78.7				

**Macrolide**	**overall**	407.1	0.0	0.0	0.0	0.0	0.0	0.0	0.0	153.1	85.2	168.8
	mph(A)	335.1								126.7	62.9	145.5
	mph(E)	11.4								7.8		3.6
	msr(E)	11.4								7.8		3.6
	erm(B)	49.3								10.9	22.3	16.1
**Quinolone**	**overall**	508.7	0.0	0.0	0.0	0.0	0.0	61.0	0.0	180.6	143.5	123.7
	crpP	0.2								0.2		
	qepA4	6.0								1.4		4.6
	qnrB1	23.3								16.6	0.4	6.3
	qnrB4	26.5								12.7	2.0	11.7
	qnrS1	362.1								128.4	132.5	101.1
	qnrS2	11.6								11.6		
	qnrS13	61.0						61.0				
	qnrVC1	10.8								2.3	8.5	
	qnrVC4	7.2								7.2		
**Rifampicin**	*ARR-3*	19.6								9.4	8.5	1.8
**Sulfamethoxazole/Trimethoprim**	**overall**	800.5	0.0	0.0	0.0	0.0	0.0	129.7	0.0	300.3	119.4	251.1
	dfrA1	43.5								6.2	8.5	28.8
	dfrA5	26.7								25.4		1.3
	dfrA12	90.6								27.1	21.5	41.9
	dfrA14	110.3						65.8		26.1	4.6	13.8
	dfrA15	1.9									1.9	
	dfrA17	42.6								27.8		14.8
	sul1	365.7								187.7	55.5	122.5
	sul2	116.7						64.0			27.4	25.3
	sul3	2.5										2.5
**Tetracycline**	**overall**	298.5	0.0	0.0	0.0	0.0	0.0	61.0	0.0	89.5	45.6	102.5
	tet(39)	1.1								1.1		
	tet(A)	234.2						61.0		66.3	22.5	84.6
	tet(B)	28.7								13.9	2.2	12.6
	tet(C)	6.5								6.5		
	tet(E)	2.3								1.7		0.6
	tet(X4)	4.8										4.8
	tmexC3	7.0									7.0	
	tmexD3	7.0									7.0	
	TOprJ3	7.0									7.0	
**Total**		**6014.0**	334.7	354.3	132.4	112.0	52.0	1173.4	277.3	1514.8	759.4	1303.7

*Japan: The downstream site of Akashi river (AKR-D), The downstream site of Mukogawa river (MUR-D); Indonesia: Bendungan Mlirip (BM), Jembatan Legundi (JL), PDAM Karang Pilang (PKP), Jembatan Rolag Gunung Sari (JRGS), PDAM Jagir (PJ); Nepal: The river near Chirayu National Hospital (CNH), The river near Marcopo-lo Medical Centre (MMC), The river near Om Hospital & Research Center (OHRC)

#RPKM: reads per kilobase per million mapped reads

Table S3. Statistical analysis of multiple comparisons at ARGs of each culture sample from river water in Japan, Indonesia and Nepal.

Level 1	Level 2	*p* -value
Japan-AKR-D	Japan-MUR-D	1.0000
Japan-AKR-D	Indonesia-BM	1.0000
Japan-AKR-D	Indonesia-JL	1.0000
Japan-AKR-D	Indonesia-PKP	1.0000
Japan-AKR-D	Indonesia-JRGS	0.6323
Japan-AKR-D	Indonesia-PJ	1.0000
Japan-AKR-D	Nepal-CNH	0.1728
Japan-AKR-D	Nepal-MMC	0.3561
Japan-AKR-D	Nepal-OHRC	0.1273
Japan-MUR-D	Indonesia-BM	1.0000
Japan-MUR-D	Indonesia-JL	1.0000
Japan-MUR-D	Indonesia-PKP	1.0000
Japan-MUR-D	Indonesia-JRGS	0.3730
Japan-MUR-D	Indonesia-PJ	1.0000
Japan-MUR-D	Nepal-CNH	0.1042
Japan-MUR-D	Nepal-MMC	0.1255
Japan-MUR-D	Nepal-OHRC	0.0458 *
Indonesia-BM	Indonesia-JL	1.0000
Indonesia-BM	Indonesia-PKP	1.0000
Indonesia-BM	Indonesia-JRGS	0.3748
Indonesia-BM	Indonesia-PJ	1.0000
Indonesia-BM	Nepal-CNH	0.0845
Indonesia-BM	Nepal-MMC	0.1728
Indonesia-BM	Nepal-OHRC	0.0488 *
Indonesia-JL	Indonesia-PKP	1.0000
Indonesia-JL	Indonesia-JRGS	0.3285
Indonesia-JL	Indonesia-PJ	1.0000
Indonesia-JL	Nepal-CNH	0.0694
Indonesia-JL	Nepal-MMC	0.1728
Indonesia-JL	Nepal-OHRC	0.0317 *
Indonesia-PKP	Indonesia-JRGS	0.2457
Indonesia-PKP	Indonesia-PJ	1.0000
Indonesia-PKP	Nepal-CNH	0.0694
Indonesia-PKP	Nepal-MMC	0.0845
Indonesia-PKP	Nepal-OHRC	0.0317 *
Indonesia-JRGS	Indonesia-PJ	0.5800

Indonesia-JRGS	Nepal-CNH	0.9990
Indonesia-JRGS	Nepal-MMC	1.0000
Indonesia-JRGS	Nepal-OHRC	0.9999
Indonesia-PJ	Nepal-CNH	0.1728
Indonesia-PJ	Nepal-MMC	0.4018
Indonesia-PJ	Nepal-OHRC	0.1065
Nepal-CNH	Nepal-MMC	0.9946
Nepal-CNH	Nepal-OHRC	1.0000
Nepal-MMC	Nepal-OHRC	0.9996

ARGs: antimicrobial resistant genes

Statistical analysis of multiple comparisons: Steel-Dwass analysis

*：p <0.05 **：p <0.01
